# β‑Cyclodextrin-Silver Nanoparticles Inclusion Complexes: Insights into Applications in Trace Level Detection (Light-Driven and Electrochemical Assays) and Antibacterial Activity

**DOI:** 10.1021/acsomega.5c03312

**Published:** 2025-06-04

**Authors:** Elisângela Gomes de Lima Oliveira, Helinando Pequeno de Oliveira

**Affiliations:** Instituto de Pesquisa em Ciência dos Materiais, 74373 Universidade Federal do Vale do São Francisco, 48902-300 Juazeiro, Bahia, Brazil

## Abstract

The inclusion complex formation of β-cyclodextrin/silver nanoparticles uses superior optical, electrical, and structural properties of AgNPs and the singular hydrophobic cage/hydrophilic external surface of the β-cyclodextrin, enabling several properties such as controlled aggregation degree, scattering of light, and adsorption of species. Consequently, several applications are favored, ranging from antimicrobial and antibiofilm agents to analyte trace detectors based on fluorescence, scattering of light, and electrochemical responses as single or combined multimode sensing elements. Herein, applications based on guest–host complexes are discussed, focusing on the potential and limitations of each technique in developing highly sensitive and low-cost templates for identification, adsorption, and quantification of different target systems. The potential of multisensing templates and electroenhanced antibacterial supports for AgNPs/β-CD incorporation is discussed as a promising strategy to reach outstanding performance for sensors and active antibacterial sensors.

## Graphical abstract

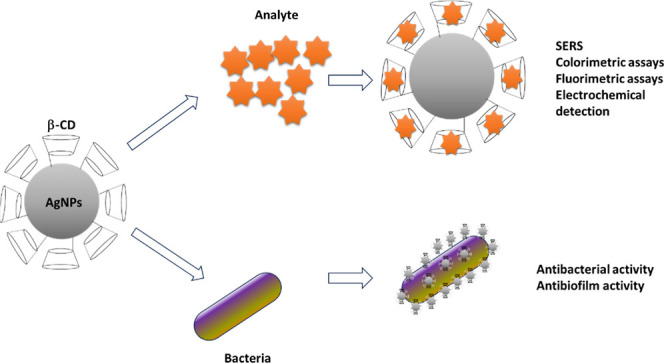

## Introduction

Cyclodextrins (CDs) are natural macromolecular oligosaccharides composed of glucopyranoside unities^1^ converted from linear to cyclic molecules under enzymatic hydrolysis.^2^ The arrangement of d-glucopyranose monomers (linked by glycosidic bonds) results in the assembly of six (α-cyclodextrin), seven (β-cyclodextrin), and eight (γ-cyclodextrin) units, providing the CD a truncated cone structure^3^ characterized by a cage and a channel complex structure.^4–7^ While the external surface of the CD is hydrophilic, the inner surface is hydrophobic, allowing the most common application as an inclusion complex,^2,3,8^ conferring improved stabilization of guests against sublimation, volatility, and oxidation.^9^ As a result, industrial applications involving cyclodextrins have been focused on their ability to encapsulate several organic compounds in their hydrophobic core.^10^

A diversity of applications has been observed for CDs in several areas, such as the food industry (inclusion and release of flavors),^11,12^ drug release,^13–15^ environmental remediation,^2,16–18^ electrochemical^19–24^ and fluorescent sensors,^25,26^ environment removal of heavy metal,^27–29^ and textile-related uses.^3,26,30–32^ All of these applications are facilitated by the formation of inclusion complexes, which are enabled by the prevailing noncovalent forces within the hydrophobic core of the CDs.^1,2,33,34^ In particular, the cavity of the most common CD (the β-CD) is 6–6.5 Å, providing a hydrophobic environment that prevails in interactions with target species through van der Waals forces, hydrogen bonding, and hydrophobic interactions.^3,35,36^ Highlighting the cyclodextrin’s biocompatibility and therapeutic potential, Almeleebia et al.^37^ reported using cyclodextrin-based nanocrystals in wound healing, exploring the dual release of rutin and thymoquinone. It is worth mentioning that biophysical methods for the characterization of bioactive molecules and inclusion complexes consider the nuclear magnetic resonance method as a gold standard platform to evaluate structure–activity relationships.^38^

Several aspects are beneficial for the application of CDs, such as using nanoreactors for metal nanoparticle synthesis due to their large number of hydrogen donors/acceptors.^5,6^ Based on this property, these structures have been successfully applied as capping agents and reducing components for AgNPs formation.^5,34^ In addition to the capping effect that results in high colloidal stability for complexed species, a reduction in the growth rate of nanoparticles is affected by the CD presence.^36^ Consequently, controllable surface-to-volume ratios are established with the advantage of the capping effect of CDs that reinforce applications based on the electrochemical detection of analytes, surface enhancement Raman scattering, fluorometric assays,^39,40^ colorimetric methods,^41^ and biological application in antibacterial and antibiofilm activities since different parameters can be monitored from the interaction of inclusion complexes and analytes such as changes in the surface plasmon resonance band, changes in color for naked eye identification, Raman spectrum, and electrochemical signature changes from processes schematically drawn in Figure 1.

**Figure 1:**
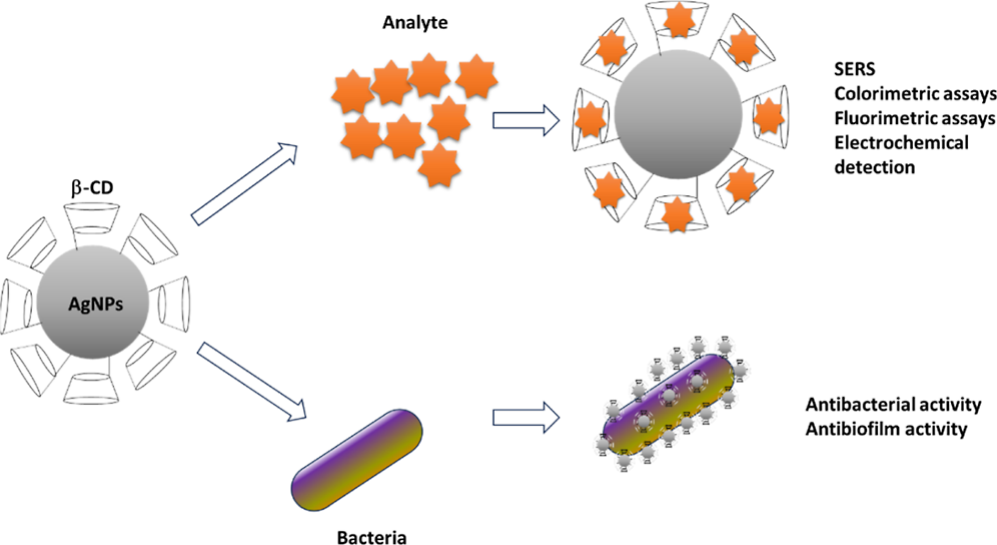
Schematic representation of the molecular disposition of the inclusion complex of β-CD and AgNPs, providing the incorporation of analytes into the hydrophobic core of the structure and the subsequent properties explored for the identification of analyte or contaminant removal.

Regarding the antibacterial activity, the Trojan horse mechanism has been attributed to bacterial carbohydrate affinity processes with AgNPs/β-CD, reinforcing the silver ion adsorption and release into membrane cells^35,42–44^ and as an antifouling coating^45^ with its characteristic low toxicity due to the capping layer of cyclodextrin^44^ characterized as an eco-friendly reducing agent^43^ disposed as pure and in association with fillers such as graphene oxide.^42,46^ The Trojan horse mechanism is explored for developing structures (agents) that can cross bacterial outer membranes, releasing toxic components after the penetration step, as observed for siderophore-antibiotics-based compounds.^47^ In particular, for silver nanoparticle-based systems, the mechanism is assigned to an initial step in which silver nanoparticles are accumulated on the membrane cell wall and disposed of as nanoparticle reservoirs.^48^ In particular, this process can be reinforced by electrostatic interaction between positively charged AgNPs and negatively charged cell walls, providing an effective cell–AgNPs interaction. Following this adhesion, the penetration of nanoparticles into the cell takes place for the following step: ionization and the release of Ag^+^ ions. The release of ions results in the formation of intracellular reactive oxygen species (ROS) and lipid peroxidation, provoking cell death.^49^

Moreover, for the electrochemical detection of analyte traces,^50,51^ the use of silver nanoparticles has also been considered as redox reporters for the detection of amyloid-β-oligomers,^51^ traces of nitroaromatic isomers,^52^ evaluation of Cu (II),^50^ and traces of ciprofloxacin,^53^ and it was based on enantio- and molecular selectivity provided by CD’s hydrophobic core.^50^ Alternatively, for the light-mediated process of trace identification, colorimetric sensors based on β-CD/AgNPs^52,54^ make possible new trends in point-of-care platforms for molecular diagnosis in response to host–guest interactions, as evidenced by shifts in characteristic absorbance peaks provoked by the aggregation steps of silver nanoparticles or changes in interaction with the target molecule, which are also observable in the naked-eye condition. Based on this concept, several templates have been assembled for applications as detectors of SARS-COV-2,^34^ encapsulation of creatinine for identifying H_2_O_2_ in urine,^54^ and identification of zidovudine^9^ and aromatic isomers.^41^ Regarding the surface Raman scattering from metal nanoparticles, the aggregation level (creating hot spots) critically affects the analyte trace detection level. The proper polarity and available size for molecular incorporation into β-CD-AgNPs cavities can be conveniently explored to enhance the surface-enhanced Raman scattering (SERS) signal in identifying diverse compounds, such as methotrexate^55^ and polycyclic aromatic hydrocarbon (PAH) compounds.^56^ The following sections will discuss each specific application for β-CD-AgNPs, considering the potential and limitations of state-of-the-art prototypes for inclusion complexes that consider integrating multisensing techniques for improved detection of analytes.

## Light-Based Techniques for the Detection of Analytes in β-CD-AgNP Compounds

### β-CD-AgNP-Based Templates for SERS Applications

SERS is an ultrasensitive analytical technique applied to identify traces of analytes,^56^ critical in areas such as therapeutic drug monitoring and identifying environmental contaminants in water.^57^ SERS signal intensity depends on the adequate entrapment of target molecules on hot spots (represented by the rough surface or colloidal dispersion of metal nanoparticles).^58^ The homogeneous distribution of gold/silver nanoparticles in the reinforcement of the SERS signal is critical; however, it is affected by the lack of selectivity from typical supports, being necessary to incorporate systems based on antibodies and molecularly imprinted polymers as a part of the strategy to minimize the aggregation level of metal nanoparticles. The use of β-CD derivatives represents an alternative to these expensive methods, providing cost-effective recognition solutions by available hydrophobic cavities, characterized as a weak Raman signal (a property that improves the signal-to-noise ratio for the resulting SERS sensors).^7,53,55,57,59–62^ Based on these aspects, different strategies have been explored to control the aggregation level of silver nanoparticles, providing the desirable selectivity, described as follows:

Hahm et al.^56^ proposed a SERS supporting template for the detection of PAH, which is based on the production of β-CD dimer—immobilized AgNP nanoparticle-embedded silica nanoparticles, as schematically drawn in Figure 2. The homogeneous dispersion of AgNPs on silica favors the creation of hot spots, enhancing the SERS signal. Moreover, the thioether-bridged dimeric β-CD immobilizes cages on AgNPs, improving their ability to capture perylene (a type of PAH). As a result, the dimeric structures of β-CD yielded a 1000-fold greater sensitivity compared to Ag@SiO_2_ systems, achieving a limit of detection (LOD) of 10^–8^ M, characterizing the potential of the hydrophobic core of CD acting as target points to receive PAH derivatives, in a process that drastically affects the pattern of scattered light.

**Figure 2:**
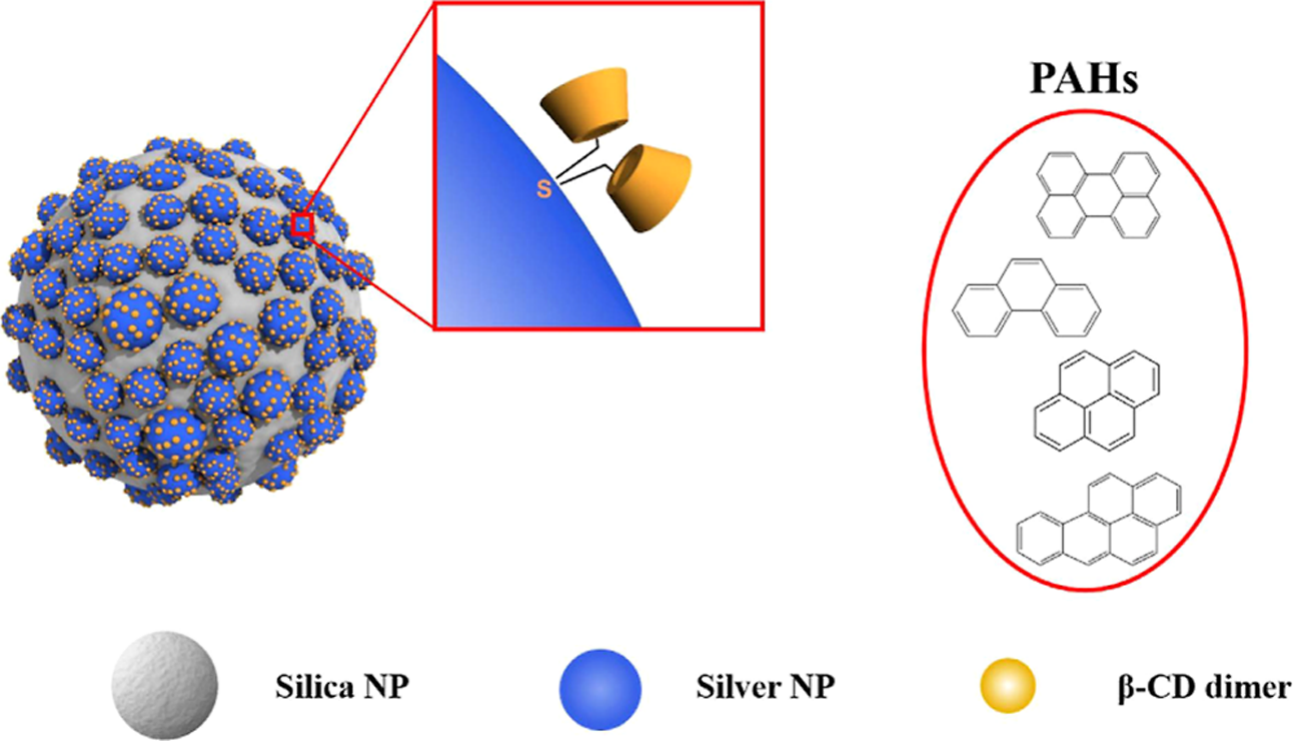
Schematic representation of β-CD dimer-immobilized AgNPs embedded in silica nanoparticles as standard hotspot structures for SERS detection of PAH derivatives. Reproduced from ref 56 with permission from Nature.

As an alternative, it has been reported^63^ the modification of β-cyclodextrins (β-CDs) with ethylamine for immobilizing species on silver nanoparticles (AgNPs) embedded in silica nanoparticles to detect flavonoids.^63^

In addition to silica-based templates, strategies based on producing gel capsules of poly­(vinyl alcohol) are employed to prevent the aggregation of immobilized Ag/β-CDs species.^64^ Bimetallic conjugates of Au core—Ag shell with β-CDs as a capping agent^4^ and structures of Ag@Fe_3_O_4_@Ag/β-CDs^65^ are some of the strategies that can be considered adequate conditions for the regular distribution of hot spots and magnetically active supports that can be externally excited for the fast and effective removal of adsorbed components. Table 1 summarizes a list of applications of β-CD-AgNPs as SERS-based sensors.

**Table 1:** Description of Experimental Systems, Corresponding Target Molecules, Detection Techniques, and Detection Limit for β-CD–AgNP-Based Systems

experimental system	target	application	limit-of-detection	ref
ethylenediamine-modified β-cyclodextrin immobilized on silver nanoparticle (NP)-embedded silica NPs	luteolin	SERS	10^–7^ M	^63^
β-cyclodextrin capped in situ onto Ag nanoparticles and encapsulated into polyvinyl alcohol	sibutramine hydrochloride	SERS	3.0 μg/mL	^64^
Ag@Fe_3_O_4_@Ag/β-cyclodextrin	butylbenzyl phthalate	SERS	1.3 mg/kg	^65^
β-cyclodextrin-modified AgNPs	anticancer methotrexate	SERS	0.3 μg mL^–1^	^55^
AgNPs modified with β-cyclodextrin	fluoroquinolone	SERS	2.9–5.8 μg/mL (urine) 0.05–0.34 μg/mL (blood plasma)	^57^
zeolitic imidazolate framework-8-wrapped Ag nanoparticles modified with β-cyclodextrin	thiacloprid and imidacloprid	SERS	1.50 nmol/L (thiacloprid) 0.83 nmol/L (imidacloprid)	^84^
β-CD-functionalized AgNPs	melamine	colorimetric detection	4.98 × 10^–6^ M	^72^
β-cyclodextrin-stabilized AgNPs	hydrogen peroxide in urine	colorimetric detection	1.47 nM	^54^
β-cyclodextrin-grafted citrate/AgNPs	riboflavin	colorimetric detection	167 nM	^73^
green AgNPs-modified β-cyclodextrin	zidovudine	colorimetric detection	42 μM	^9^
β-cyclodextrin-stabilized AgNPs	Ni^2+^ ion in water	colorimetric detection	33.30 ppb	^85^
β-cyclodextrin-functionalized AgNPs	mercury cation (Hg^2+^) and sulfide anion (S^2–^)	colorimetric detection colorimetric	37.50 × 10^–9^ mol/dm^3^ (Hg^2+^ ions) 0.90 × 10^–9^ mol/dm^3^ (S^2–^ ions)	^86^
molecularly imprinted polymer-coated pencil graphite modified with AgNPs	cortisol	electrochemical detection	0.214 nM	^83^
reduced graphene oxide and β-cyclodextrin	methadone in human biofluids	electrochemical detection	333.33 nM	^80^
AgNPs-embedded conductive hydrogel	hydroquinone	electrochemical detection	0.12 μM	^82^
poly(β-CD)-AgNPs	silibinin	electrochemical detection	0.0103–10.3 μM (linear range)	^81^
silver selenide anchored on β-CD/reduced graphene oxide	azithromycin	electrochemical detection	0.0045 μM	^79^
Ag nanoparticles decorated on cadmium sulfide nanowires/reduced graphene oxide	acyclovir	electrochemical detection	3.3 nM	^78^
N-CQDs/AgNPs/β-CD nanomaterials	Fe(II) and Fe(III) irons	electrochemical detection	0.2 mM (Fe (II)) 0.033 mM (Fe (III))	^77^
silver–copper oxide core–shell nanoparticles/β-cyclodextrin-functionalized SWCNT	4-chloro-3-methylphenol	electrochemical detection	1.4 nM	^76^
silver nanoparticles modified with aminated carbon nanotubes	phenylalanine enantiomers	electrochemical detection	4.62 μM (d-Phe) 5.23 μM (l-Phe)	^75^
β-cyclodextrin-modified silver nanoparticles	ciprofloxacin	electrochemical detection	0.028 nM	^53^
silver nanoparticles-β-cyclodextrin-graphene nanocomposites	guanine and adenine	electrochemical detection	0.09 mM (guanine) 0.15 mM (adenine)	^74^

### Colorimetric Sensors

Colorimetric and naked-eye detection of analytes from nanostructured systems are simple, low-cost, and rapid methods for the point-of-care identification of specific compounds. The change in the characteristic color of compounds in solution or complete suppression for the visual or spectroscopic identification of analyte in solution is crucial for the colorimetric detection of contaminants,^66,67^ in which the control of the plasmonic response is critically necessary.

A plasmon is a collective oscillation of free electrons in a noble metal (typically, silver and gold). For metal nanoparticles (with a size comparable to the wavelength of light), the particles’ free electrons participate in the collective oscillation as localized surface plasmons. Localized surface plasmon resonance (LSPR) events result in sharp absorption and scattering peaks strongly affected by several factors such as nanoparticle size, shape, and aggregation state.^68^ While absorption dominates LSPR extinction for the smallest nanoparticles, the scattering prevails at increasing diameter,^69^ making it possible to establish a direct relationship between LSPR and the size of nanoparticles. Consequently, a shift in the LSPR toward a longer wavelength under aggregation and increasing size of nanoparticles is observed, with silver nanoparticles being preferable to AuNPs due to their higher extinction coefficient.^70,71^ The electrostatic interaction of oppositely charged species is considered the most common mechanism for a more substantial aggregation of AgNPs in the presence of analytes. For example, under the influence of negatively charged citrate-stabilized AgNPs or through the formation of an inclusion complex provided by β-CD, several processes can be added to the standard electrostatic interaction, such as van der Waals forces and hydrogen bonding, thereby optimizing the interaction with the analyte.^72^

Potential prototypes for detecting analytes in colorimetric assays are reported as follows: John Xavier et al.^72^ reported detecting melamine, in which the characteristic positively charged groups (NH_2_) interact with the host–guest inclusion centers in β-CD-functionalized AgNPs. The incorporation of sensing elements has also been reported for creatine-assisted detection of H_2_O_2_, in which the interaction between the cavity of β-CD and creatinine enhances the selectivity of the resulting inclusion complex, achieving a LOD of 1.47 mM.^54^

Ma et al.^73^ highlight the relevance of hydrogen bonding between riboflavin and β-CD, aiming to enhance the aggregation of AgNPs and, consequently, the shift in the SPR band^73^ that is provoked by the reduction in the distance between dispersed silver nanoparticles (a crucial factor in identifying specific target molecules in solution) which is facilitated by the available hydrophobic cavities of β-CDs. This process is illustrated in Figure 3, which schematically represents the interaction between zidovudine and β-CDs with the reduction in the distance between adjacent plasmonic nanoparticles, applied in aggregation-based processes.^9^

**Figure 3:**
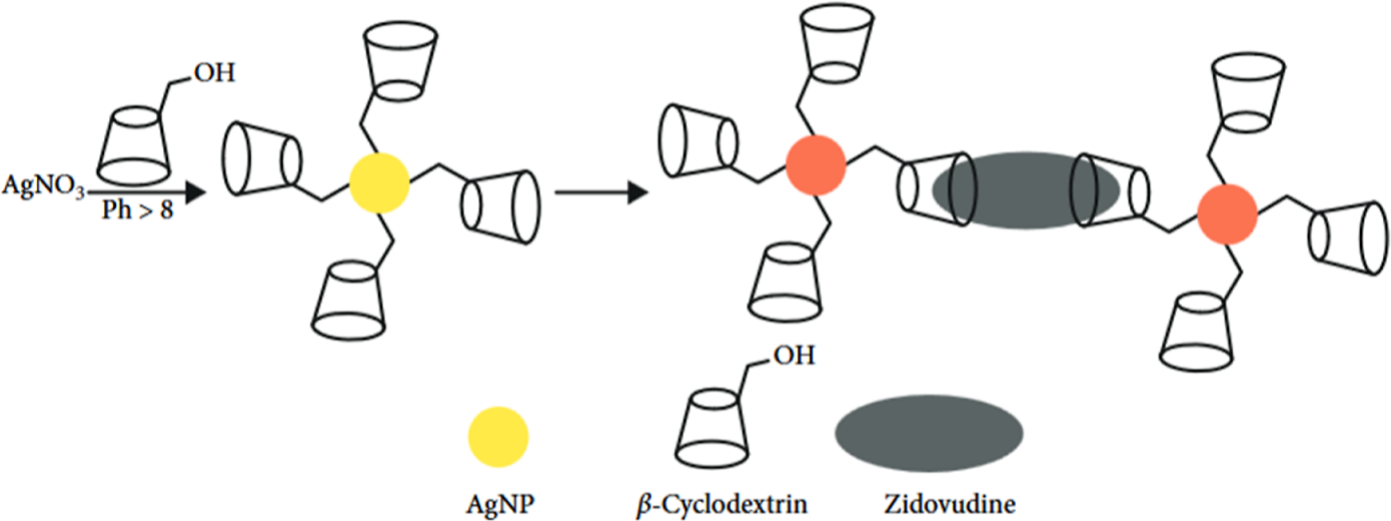
Schematic diagram of β-CD on AgNPs as a colorimetric template-based structure for detecting zidovudine. Reproduced from ref 9 with permission from Wiley.

### Electrochemical Sensors

The development of electrochemical sensors for the detection of analytes based on β-CDs and AgNPs has been favored by the incorporation of carbon-based supports (graphene,^74^ aminated multiwalled carbon nanotubes,^75^ single-walled carbon nanotubes,^76^ N-doped carbon quantum dots,^77^ and reduced graphene oxide^78–80^ that are dispersed on electrodes acting as potential amplifiers for electrochemical signals). Moreover, there are drawbacks to using carbon-based structures regarding the aggregation of nanotubes and the restacking of platelets. The anchoring of silver nanoparticles on carbon templates circumvents these aggregation steps, providing the advantage of their intrinsic high conductivity, chemical stability, and high-surface area.^78^ Moreover, the functionalization of AgNPs with β-CD offers a high density of available sites, improving the selectivity of arrangement for specific molecules (such as guanine and adenine),^74^ ciprofloxacin,^53^ chiral selection of phenylalamine,^75^ chlorophenols,^76^ Fe (II) and Fe­(III) ions,^77^ acyclovir,^78^ azithromycin,^79^ silibinin,^81^ hydroquinone,^82^ methadone,^80^ and cortisol.^83^ Details about active materials and detection limits are summarized in Table 1.

In addition to using carbon-based templates (CNTs and graphene), the impregnation of AgNPs into conductive hydrogels^82^ and the following electropolymerization of β-CDs are possibilities to consider as alternatives to carbon-based substrates. In common, the detection of analyte traces in hydrogel-based templates by differential pulse voltammetry is also amplified and favored by hydrogen bonds between β-CD and the specific molecule in its singular structure.

## Multimode Sensing Techniques

Multimode sensing methods are promising strategies to advance the frontiers of knowledge through the combination of conventional detection methods. They circumvent drawbacks from single-mode sensing strategies while incorporating advantages from self-correction, self-validation, lower trace-level detection, enhanced reliability and stability, selectivity, and reduction in false positives.^87–89^

With this aim, dual-mode sensing methods combine electrochemical and colorimetric,^87,88^ SERS and colorimetric,^89–92^ and SERS and fluorescent platforms.^93^ The production of core–shell structures with the incorporation of probe molecules and the induced electrochemical reactions to potentialize the detection of traces of analytes are some of the strategies that favored the consorted operation of detection methods.

Based on these strategies, Forzani et al.^87^ reported the combined electrochemical reaction for colorimetric detection of the reaction products. In particular, combining electrochemical and colorimetric methods mitigates potential interference from real samples in colorimetric detection.^88^ On the other hand, the combination of SERS and colorimetric platforms can make use of several core–shell combined materials, such as rhodium nanocores coated by AgNPs, optimizing both plasmonic behavior and hot spots for SERS, reaching LOD for mycotoxin of 4.21 pg/mL;^89^ regarding the detection of Shiga toxin type (II), a LOD of 2.6 pg/mL (colorimetric assay) and 0.82 ng/mL (SERS) is reported;^91^ while for pesticide chlorpyrifos, the value for LOD is 1 ppb (AgNPs).^92^

Yang et al.^93^ used probe molecules with mutual activity in dual sensing modes, using rhodamine 6G as a mutual fluorescent and Raman probe for an AgNPs-β-CD template. In the presence of R6G molecules, the interaction of the probe molecule with the hydrophobic cavity of β-CD results in fluorescence quenching. The Raman technique appears as a secondary technique to distinguish the nature of the analytes under investigation: amantadine and cholesterol. While one cholesterol forms an inclusion complex by two Ag-β-CD nanoparticles, just one nanoparticle is necessary to bind with amantadine. Consequently, some degree of aggregation is favored by cholesterol-inducing hot spot formation. An enhancement in the Raman signal is observed, making it possible to distinguish between analytes that present comparable responses in colorimetric assays but distinguishable responses in the Raman response. Figure 4 summarizes the process of the inclusion complex assembly and the association of sensing mechanisms into the desirable identification of different analytes by complementary information provided by SERS and colorimetric assays.

**Figure 4:**
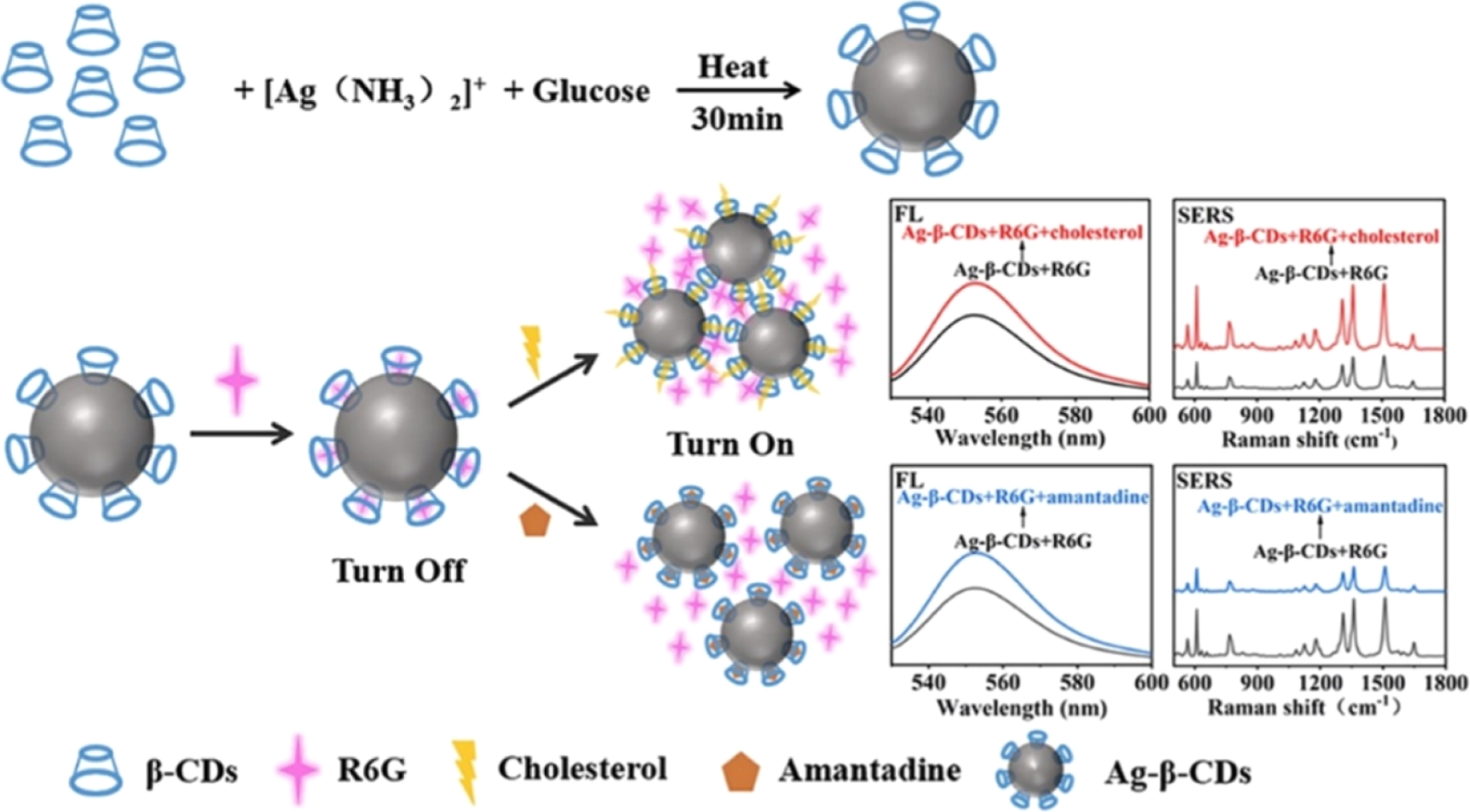
Schematic representation of the assembly process of cyclodextrin and reduced AgNPs as templates for fluorescent and SERS detection of cholesterol and amantadine. Reprinted from *Chinese J. Anal. Chem*, 53, Z. Yang, H. Lv, N. Zhang, X. Ju, Z. Zhang, X. Cui, Y. Tian, D. Song, SERS and fluorescence dual-mode sensing strategy based on competitive host–guest interaction for cholesterol and amantadine detection, Page 100480, Copyright (2025), with permission from Elsevier.

In addition to the promising use of dual-sensing platforms for the detection of traces of analytes in real samples by the complementary information provided by the measured signal, the integrated information has been considered as the input for the machine learning model, demonstrating to be a promising strategy in which *k*-nearest neighbor and artificial neural network returned outstanding LOD (37-fold increase than single method of detection),^89^ indicating that intelligent methodologies based on machine learning analysis applied in classification are critical for adequately classifying and quantifying contaminants in complex samples.

### Antibacterial Activity of β-CD-AgNPs

The major challenge for alternative antibacterial prototypes is overcoming the adaptive mechanisms of multidrug-resistant (MDR) bacteria, which have been identified as a critical risk factor for hospital morbidity and mortality^94–99^ associated with the scarcity of new conventional antibiotics. Despite the intrinsic toxicity of silver nanoparticles and ions, these nanostructures have been regarded as promising building blocks for circumventing MDR processes.^94–97^ The use of inclusion complexes in supramolecular assemblies of host–guest compounds has been considered a strategy to minimize the toxicity of nanoparticles, which offer the advantages of localized nanoparticle activity, thereby optimizing therapeutic conditions by administering a lower dose of the active antibacterial compound.^98^ The formation of inclusion complexes of cyclodextrins enables the encapsulation of AgNPs^99^ and active molecules that can be released into the desirable target. Based on this condition, conventional drugs such as ketoconazole, with potential antifungal and antibacterial activities, are successfully entrapped on β-CDs to achieve the corresponding activity using a lower drug content.^99^ The general mechanisms for the antibacterial activity of AgNP-based compounds are considered at three levels, described as follows:

First, releasing Ag^+^ ions from a supporting template under interaction with the bacterial cell membrane affects bacterial permeability and transport systems, which results in polarization changes in the cytoplasmic membrane. As a consequence of the progressive generation of ROS from Ag^+^ ions, the cessation of respiratory processes occurs, resulting in cell kill.^100^ For the controlled activity of silver nanoparticles in bacterial cells, a critical point to be considered is the nature of the encapsulating agent, which affects not only the release ratio but also the toxicity and adhesion degree of AgNPs in bacterial cells.

The most common supports for silver nanoparticles and AgNPs/CD complexes are hydrogel-based systems, electrospun nanofibers, and cotton textile-based templates. The general process for the interaction of AgNPs loaded in hydrogels with bacterial cells is based on the electrostatic interaction between the hydrogel and negatively charged moieties in the cell membrane, which is followed by the attachment of AgNPs/their aggregation and the disruption of the cell wall.^100^ The increase in the hydrophilic strength and swelling of the hydrogel is explored for complexes of β-CD, which is favored by the homogeneous dispersion of AgNPs into the matrix,^101^ described as follows.

### Hydrogel-Based Support for AgNPs Incorporation

Hydrogels are promising soft materials assembled into a 3D cross-linked network rich in hydrophilic groups, resulting in a structure that becomes swollen in water due to its high water density, which maintains its shape and enables applications in biomedicine due to the potential biocompatibility, biodegradability, and environmentally friendly behavior from different hosts applied in hydrogels such as alginate, gelatin, chitosan, agar, and carrageenan.^102–104^

Based on these properties, several advantages of hydrogel-based release systems can be considered, including green chemistry principles that leverage the benefits of reduced AgNPs and phytochemical compounds on the hydrophobic core of CDs.

Dai et al.^100^ describe an environmentally friendly method for synthesizing a functionalized hydrogel using AgNPs and natural polymer-based materials, which applies the technique of plant-mediated AgNPs.^105–108^ With this aim, the peanut red skin extract is stabilized by cyclodextrin and applied as a reducing agent for AgNP production, utilizing the inclusion mechanism between β-CD derivatives and peanut red skin, which enables the use of their antioxidant and antibacterial properties in compounds with promising applications in wound healing processes. Figure 5 summarizes the overall process of compound preparation, with the incorporation of the peanut red skin extract into the hydrophobic cage of hydroxypropyl-β-CD (Figure 5A), explored in the reduction of silver nanoparticles (Figure 5B), for the following adhesion of silver nanoparticle aggregates into bacterial cell walls.

**Figure 5:**
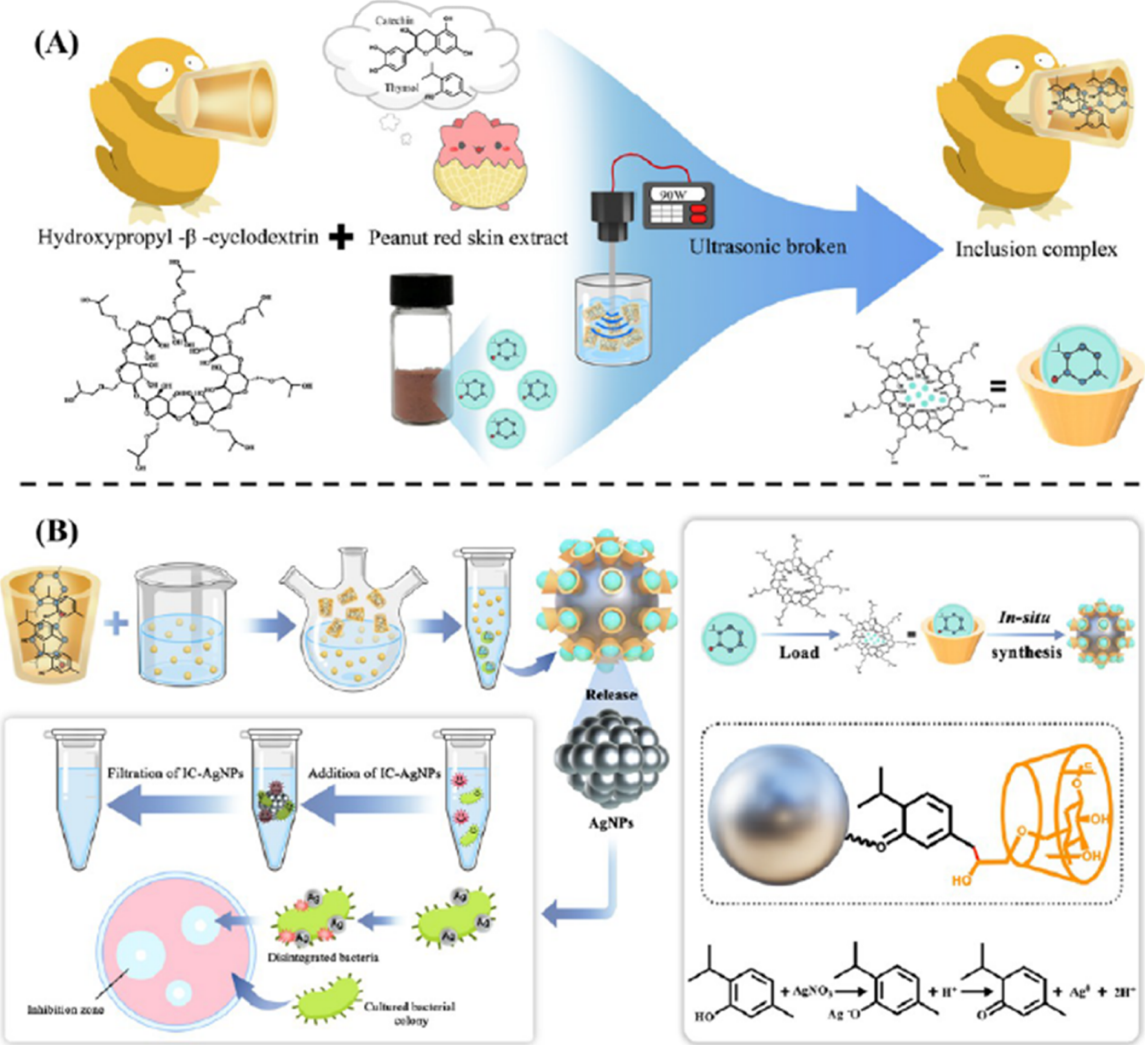
(A) Schematic representation of the formation of an inclusion complex for the green synthesis of AgNPs and (B) proposed mechanism for releasing AgNPs from the inclusion complex and subsequent antibacterial activity. Reprinted with permission from ref 100 Copyright 2024 American Chemical Society.

The authors reported a rapid disruptive effect on the outer and cytoplasmic membranes of Escherichia coli within 3 min of using this experimental system as an antibacterial agent.

Alternatively, Gupta et al.^33^ reported using curcumin as a natural reducing agent to produce AgNPs incorporated into hydroxypropyl-β-cyclodextrin and loaded into a bacterial cellulose hydrogel. The hydrophobicity of curcumin, circumvented by microencapsulation in β-CDs, and the good cytocompatibility provided the material with promising antibacterial activity.

### Electrospun Nanofiber-Based Supports for AgNPs/ β-CD Compound Incorporation

Electrospun nanofibers have been successfully incorporated into several nanotechnology-based applications,^109–111^ spanning areas that range from adsorption prototypes^112,113^ to energy-related systems^114–116^ due to their characteristic tunable porosity and ease of incorporating additives. The standard experimental setup for producing electrospun fibers involves applying a high electric field to a spinneret in a syringe containing a polymeric solution under fixed pressure and positioning the syringe a few centimeters from a grounded target.^117^ Under a voltage level in the order of 5 kV–10 kV, the competition between the surface tension on the metallic tip and the electrostatic force in the direction of the target results in a transition zone (Taylor’s cone) and the formation of a flighting jet with the following evaporation of the solvent and the deposition of a nanofiber net on the grounded collector. As a result of this multivariate process, the morphology, surface, and thickness of the resulting fibers can be controlled by adjusting a combination of environmental conditions, the distance of the spinneret to the target, the voltage, humidity, and the flux of the polymeric solution.

In particular, incorporating β-CD/Ag NPs into electrospun fibers is beneficial for several reasons,^118^ based on the control of surface area, porosity, and wettability of the resulting structures that are critical parameters for the controlled release of active antibacterial agents (Ag^+^ ions or combined Ag NPs/antibiotics). The control of the contact angle to achieve a desirable level of hydrophobicity favors its application in wound dressings.^119^

Combining silver sulfadiazine (SSD) and β-cyclodextrin (β-CD) into polycaprolactone-based electrospun fibers results in minimal degradation in water. It provides adequate conditions for controlled diffusion along with the fibers.^119^ The inclusion complex between SSD and β-CD is also explored in poly­(vinyl alcohol)-based nanofibers.^120^

Moreover, alternative green-based strategies have been employed to reduce AgNPs to electrospun fibers. Nthunya et al.^121^ describe the UV photochemical reduction of nanoparticles, eliminating the need for harsh reagents. Alternatively, Khan et al.^122^ describe methods based on the adsorption of AgNPs into electrospun fibers by ultrasonication and hydrothermal approaches.

In addition to polycaprolactone, poly­(vinyl alcohol), cellulose acetate,^61,121,123^ and cationic polymers,^124^ structures based on hydroxypropyl-β-CD are also explored for electrospun templates for integration with AgNPs. Celebioglu et al. reported the use of hydroxypropyl-β-cyclodextrin as both a reducing agent and a catalyst for the formation of AgNPs with the incorporation of HP-β-CD into a mixture of DMF and water, resulting in fibers with a desirable diameter.^61^

Figure 6 shows the general scheme for preparing the inclusion complex solution and the following procedure of the electrospinning production of fibers decorated with AgNPs as active antibacterial agents.^61^ As can be seen, the structure of HP-β-CD (shown in Figure 6a) reduces Ag^2+^ into Ag^0^ at alkaline conditions under stirring (Figure 6b), reaching a dark brown color change. After the electrospinning procedure (Figure 6c), the HP-β-CD membrane loaded with AgNPs is obtained (Figure 6d).

**Figure 6:**
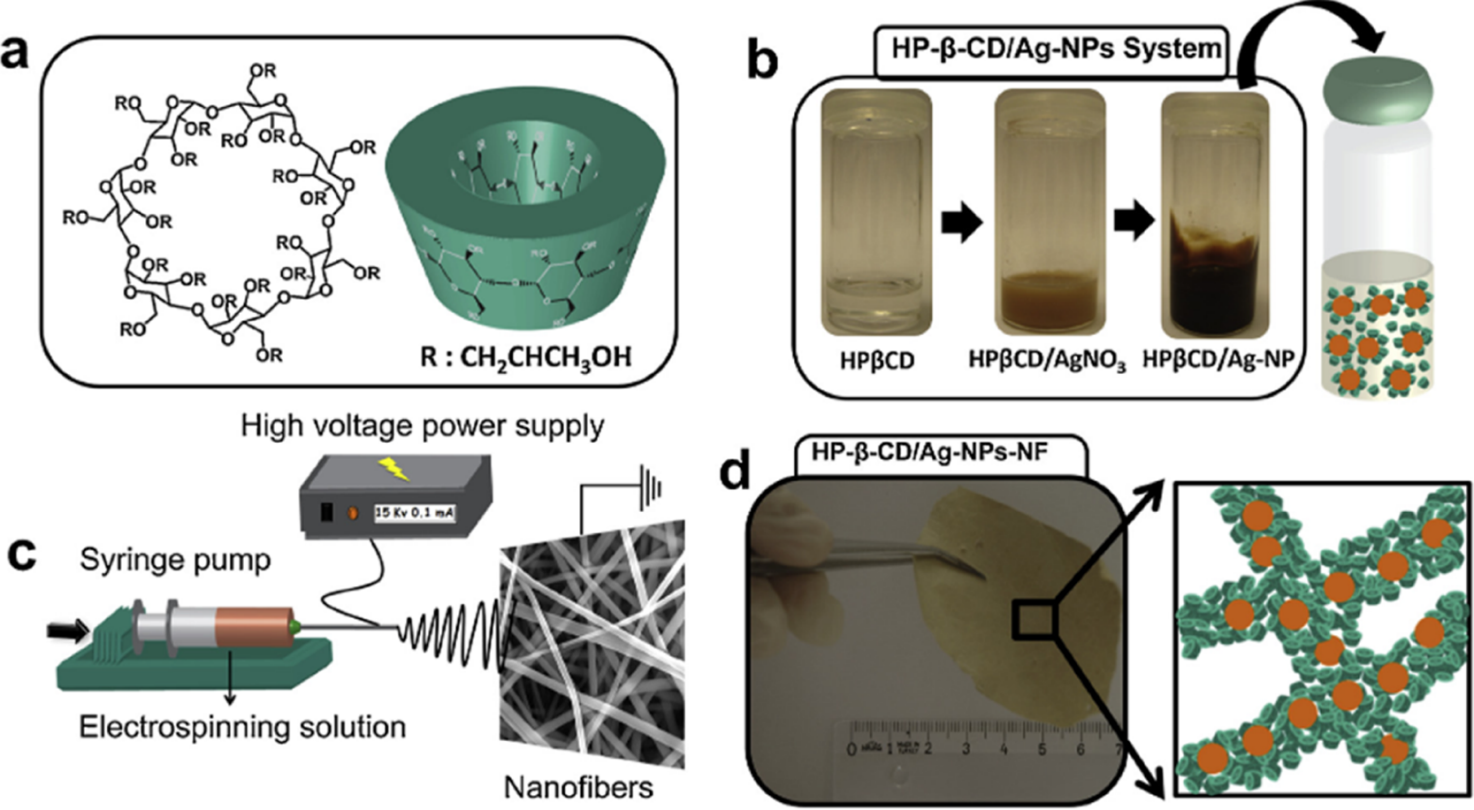
(a) Chemical structure of HP-β-CD, (b) optical images of the solution before and after the reduction of silver nanoparticles, (c) experimental setup for the electrospinning procedure, (d) optical photograph of resulting electrospun fibers. Reprinted from Carbohydrate Polymers, 207, A. Celebioglu, F. Topuz, Z.I. Yildiz, T. Uyar, One-step green synthesis of antibacterial silver nanoparticles embedded in electrospun cyclodextrin nanofibers, Pages 471–479, Copyright (2019), with permission from Elsevier.

### Cotton Fiber-Impregnated Supports

The production of high-performance, value-added cotton fabrics has focused on chemical modification to acquire innovative functionalities, including electrical conductivity, self-cleaning properties, antibacterial response, and UV protection, while preserving the intrinsic properties of the pure fabric, such as wearability, washing stability, tensile strength, and comfort.^125–128^

Moreover, the acquired properties must be retained under repeated cycles of washing and tensile efforts since desirable effects (such as electrical conductivity and antibacterial activity) are critical for applications in triboelectric nanogenerators (TENGs),^129–131^ supercapacitors,^132–134^ and wound dressing systems with the prolonged action of active components.^135,136^ Regarding incorporating silver nanoparticles into the fabric,^137^ there are *in situ* and *ex situ* methodologies for reducing nanoparticles, which are described below.

One of the most critical steps for adequate bonding of the active nanoparticles to the cotton fabrics is the cross-linking process. As a standard reagent, ethylenediaminetetraacetic acid (EDTA) has been considered a cross-linking agent^138^ and applied in the fixation of sulfated β-cyclodextrin (β-cyclodextrin chemically treated with sulfuric acid) for the subsequent incorporation of silver nanoparticles which interact with S-β-CD for the formation of inclusion complexes.^3^

Alternatively, Hebeish et al.^139^ proposed a new approach in which monochlorotriazinyl-β-cyclodextrin, grafted with acrylic acid, is applied to react with cotton for the incorporation of silver nitrate and the *in situ* reduction utilizing the copolymer as a reducing agent,^139^ avoiding the use of more aggressive reducing agents, such as sodium borohydride. Another possibility is based on the mutual reduction and graft polymerization of pomegranate-shaped silver nanoparticle compounds,^140^ resulting in aggregates of 500 nm by the wrapping formation. Regarding the requisites of cyclability and retention of the antibacterial activity after 50 washing procedures, Atav et al. reported the production of silver cyclohexane monocarboxylate and β-cyclodextrin.^141^ The authors applied it to cotton fabrics via the pay-dry method and noted the prolonged retention of antibacterial activity after multiple washing cycles, which is a desirable property for modified textiles. Table 2 summarizes the description of the experimental system and the corresponding antibacterial activity for all of the above-reported applications.

**Table 2:** Experimental Prototypes and Antibacterial Activity for β-CD–AgNP–Based Systems

experimental system	antibacterial activity	reference
hydroxypropyl-β-cyclodextrin	*E. coli* and *Staphylococcus aureus*	^61^
polyethyleneimine-β-CD-silver nanoparticles	Methicillin-resistant *S. aureus* and *E. coli*	^124^
β-cyclo-dextrin/cellulose nanofibers embedded with silver and silver/iron nanoparticles	*Bacillus cereus* and *E. coli*	^121^
cellulose acetate (CA) as the matrix and dimethyloxallyl glycine (DMOG) and silver nanoparticles (Ag-NPs) as the drug-loading component	*E. coli* and *Bacillus subtilis*	^123^
electrospun poly(ε-caprolactone) matrices with silver sulfadiazine complexed with β-cyclodextrin	*Staphylococcus epidermidis*, *E. coli* and *K. pneumoniae*	^119^
electrospun nanofibers of PVA containing SSD/CD inclusion complexes	*E. coli* and *S. aureus*	^120^
cotton textile modified with β-CD/AgNPs cross-linked with EDTA	*S. aureus* and *E. coli*	^138^
sulfated β-cyclodextrin (S-β-CD)	*S. aureus* and *E. coli*	^3^
cyclodextrin grafted with acrylic acid AA, CD-g-PAA	*S. aureus* and *E. coli*	^139^
silver cyclohexane monocarboxylate and β-cyclodextrin inclusion complexes	*S. aureus*, *B. subtilis*, and *Pseudomonas aeruginosa*	^141^

As observed, the requisites for the controlled release of Ag^+^ ions depend on adequate bonding with the releasing matrix. Another critical aspect to be considered is the possibility of using an external stimulus to reduce the concentration of the active antibacterial agent while preserving the antibacterial activity, as observed in electroenhanced assays.

### Electroenhanced Antibacterial Coatings Based on AgNPs

Several alternative strategies based on electroenhanced antibacterial coatings have been considered to potentialize the antibacterial activity of compounds, such as from the association of active nanoparticles with electrically active microenvironments, as reported by Moreira et al.^142^ that used a piezoelectric template (poly­(vinylidene fluoride-*co*-trifluoroethylene)) with AgNPs and the conversion of the mechanical energy into an electrical stimulus to improve the antibacterial activity of the AgNPs since the use of low-frequency electric fields proved to be an effective strategy to enhance the AgNPs antibacterial and antibiofilm activities.^143^ Alternatively, electrically conducting polymers such as polypyrrole^144^ and poly­(hydroxymethyl 3,4-ethylenedioxythiophene): polystyrene sulfonate (PEDOT-MeOH: PSS)^145^ can be successfully applied in corresponding assays. Gomez-Carretero et al.^145^ reported the influence of a conducting polymer layer coated by AgNPs and the influence of an external electric field (5 Hz, 4 V_pp_) on the antibacterial and antibiofilm activities of the resulting material (increase in the release rate of Ag^+^ ions).

Lastly, for applications involving inclusion complexes, additional experiments and functionalization strategies must consider long-term storage and harsh environmental conditions on the performance of the resulting nanostructures. Promising results have been reported for the use of β–CD-AgNP templates for SERS detection of analytes in terms of long-term storage (Yang et al. reported the determination of 6-mercaptopurine after long-term storage of 45 days^146^ while Ma et al. observed good retention in the stability of supports after 4 weeks of storage^147^).

## Conclusions

The use of βCD-AgNPs has been progressively explored in developing trace sensors and antibacterial compounds due to their potential for forming inclusion complexes and sensing properties/release of Ag^+^ ions from nanostructures. The big challenge for these nanostructures is the optimized activity, regarding the LOD and antibacterial activity. In both conditions, the consorted association of methods appears as a promising strategy to reach the outstanding performance. For trace-level detectors, multimode sensing can circumvent limitations from each technique and, if explored in an integrated view, as observed for machine learning interpretative assays, can be considered as a strategy to evaluate the presence of traces in real samples, with minimal interference of contaminants due to the high reported selectivity. In the same direction, the antibacterial activity of AgNP-based complexes is favored by incorporating material into electro responsive material that can control the overall antibacterial response under pulsed and low voltage level excitation. These findings reveal the essential integration of the design of experimental prototypes with techniques of evaluation of the interference level in the detection and actuation of βCD-AgNP-based inclusion complexes. As perspectives for the migration from lab scale to industrial conditions, a complete assessment of the bioeffects must be combined with adequate integration into miniaturized platforms, focusing on advancing point-of-care analytical tools. Special attention must be given to using smartphones as processing units integrated with wireless technologies and into wearables. Combining low-cost routes with eco-friendly methods for the massive production of inclusion complexes represents a critical strategy to reach the industrial production scale with minimal environmental impact.

## Data Availability

The data used in this study are available upon request
